# Establishment of a Transplantation Model of PDAC-Derived Liver Metastases

**DOI:** 10.1245/s10434-024-15514-3

**Published:** 2024-06-13

**Authors:** Benedetta Ferrara, Erica Dugnani, Antonio Citro, Marco Schiavo Lena, Paolo Marra, Paolo Riccardo Camisa, Martina Policardi, Tamara Canu, Antonio Esposito, Claudio Doglioni, Lorenzo Piemonti

**Affiliations:** 1 Diabetes Research Institute, IRCCS San Raffaele Scientific Institute, Milan, Italy; 2 Pathology Unit, Pancreas Translational and Clinical Research Center, IRCCS San Raffaele Scientific Institute, Milan, Italy; 3 Experimental Imaging Center, IRCCS San Raffaele Scientific Institute, Milan, Italy; 4 Department of Radiology, ASST Papa Giovanni XXIII Hospital, University of Milano Bicocca, Bergamo, Italy; 5 Vita-Salute San Raffaele University, Milan, Italy

## Abstract

**Background:**

The highly metastatic nature of pancreatic ductal adenocarcinoma (PDAC) and the difficulty to achieve favorable patient outcomes emphasize the need for novel therapeutic solutions. For preclinical evaluations, genetically engineered mouse models are often used to mimic human PDAC but frequently fail to replicate synchronous development and metastatic spread. This study aimed to develop a transplantation model to achieve synchronous and homogenous PDAC growth with controlled metastatic patterns in the liver.

**Methods:**

To generate an orthotopic PDAC model, the DT6606 cell line was injected into the pancreas head of C57BL/6 mice, and their survival was monitored over time. To generate a heterotopic transplantation model, growing doses of three PDAC cell lines (DT6606, DT6606lm, and K8484) were injected into the portal vein of mice. Magnetic resonance imaging (MRI) was used to monitor metastatic progression, and histologic analysis was performed.

**Results:**

Orthotopically injected mice succumbed to the tumor within an 11-week period (average survival time, 78.2 ± 4.45 days). Post-mortem examinations failed to identify liver metastasis. In the intraportal model, 2 × 10^5^ DT6606 cells resulted in an absence of liver metastases by day 21, whereas 5 × 10^4^ DT6606lm cells and 7 × 10^4^ K8484 cells resulted in steady metastatic growth. Higher doses caused significant metastatic liver involvement. The use of K8484 cells ensured the growth of tumors closely resembling the histopathologic characteristics of human PDAC.

**Conclusions:**

This report details the authors’ efforts to establish an “optimal” murine model for inducing metastatic PDAC, which is critical for advancing our understanding of the disease and developing more effective treatments.

**Supplementary Information:**

The online version contains supplementary material available at 10.1245/s10434-024-15514-3.

Pancreatic ductal adenocarcinoma (PDAC) remains one of the most challenging malignancies, holding the position as the seventh leading cause of cancer-related mortality worldwide.^1^ Despite advancements in medical research and therapeutic strategies, the prognosis for PDAC patients continues to be dire, with a 1-year survival rate of 24% and a 5-year survival rate declining to 9%.^2^ This bleak outlook is largely due to the asymptomatic nature of the disease in its early stages, leading to late diagnoses, when therapeutic options are limited and less effective.^3^

The early detection of PDAC is hindered by several factors, including the non-specificity of tumor markers and the physical inaccessibility of the pancreas for routine examination, which collectively contribute to the delayed identification and treatment of this cancer.^4^ In the majority of cases, PDAC is diagnosed at an advanced stage, characterized by significant tumor metastasis, primarily to the liver, but also to the peritoneum and lungs.^5,6^ The aggressive metastatic behavior of PDAC, even when tumors are relatively small,^7^ underscores the urgency for more effective therapeutic interventions.^8^

Currently, the therapeutic landscape for metastatic PDAC is dominated by chemotherapy, radiotherapy, and immunotherapy. However, these approaches rarely yield substantial improvements in patient outcomes.^9^ This underscores the critical need for innovative treatment strategies and the importance of preclinical models that accurately represent the human condition to facilitate the development of such interventions. In response to this need, genetically engineered mouse models (GEMMs) have been extensively used to mimic human PDAC, offering invaluable insights into the complex biology of the disease, including its genetic and molecular underpinnings and interaction with the tumor microenvironment.^4^ Despite their contributions, these models often fall short in replicating the synchronous development and metastatic dissemination characteristic of human PDAC,^10^ limiting their utility in evaluating novel therapeutic approaches.

To bridge this gap, we have developed and characterized some transplantation models of PDAC, aiming to achieve synchronous, homogeneous tumor growth with a controlled metastatic pattern, particularly focusing on liver metastases.^11^ By injecting PDAC-derived cell lines into the portal vein of autologous immunocompetent mice, we used magnetic resonance imaging (MRI) to monitor metastatic progression and performed histologic examinations to assess the resemblance of these metastases to human PDAC. This report details our efforts to establish an “optimal” murine model for inducing metastatic PDAC, which is critical for advancing our understanding of the disease and developing more effective treatments.

## Materials and Methods

### Cell Lines

The murine pancreatic adenocarcinoma cell lines, K8484 and DT6606, were generously provided by Professor Paola Cappello from the Department of Molecular Biotechnology and Health Sciences, Turin, Italy. Whereas K8484 derives from spontaneous tumors generated in the KPC mice model (LSL-KrasG12D/+; LSL-Trp53R172H/+; Pdx-1-Cre), DT6606 derives from spontaneous tumors generated in the KC mice (LSL-KrasG12D/+; Pdx-1-Cre). Both cell lines were previously tested and authenticated.^12,13^ These cell lines were cultured in RPMI-1640 medium enriched with 10 % fetal bovine serum (FBS), 2 mmol of glutamine, and antibiotics (all from Thermo-Fisher Scientific, Waltham, MA, USA, and Sigma-Aldrich, St. Louis, MO, USA), following standard cell culture protocols.

### Mouse Strain

Both male and female C57BL/6 mice, age 8 weeks and weighing between 20 and 22 g, were procured from Charles River Laboratories Italia Srl, Lecco, Italy. These mice were housed under controlled conditions, with free access to food and water, at the San Raffaele Scientific Institute’s animal facility and preclinical imaging facility in Milan, Italy. Regular health monitoring was performed thrice weekly.

### Ethics Statement

This study adhered strictly to ethical guidelines, with approval from the Animal Care and Use Committee of the San Raffaele Scientific Institute (permit nos. 559 and 896). Surgical interventions were performed with the mice under anesthesia, induced with a combination of ketamine/xylazine (100 mg/kg), and imaging procedures used general inhalation anesthesia with isoflurane. The end points of the study included humane euthanasia by cervical dislocation based on clinical symptoms or for histologic analysis.

### Orthotopic Injection of PDAC Cells

To create orthotopic pancreatic tumors, the DT6606 cell line was selected, and the procedure was performed using a previously defined protocol.^14^ The mice were anesthetized and positioned supinely for performance of a small abdominal incision, through which the pancreas was exposed. Cell suspensions prepared in ice-cold Matrigel without growth factors (25 % concentration, Corning, Somerville, MD, USA) were injected into the pancreatic head using a 29-gauge needle, ensuring that the cells were delivered accurately under microscopic guidance. After the injection, the incision was sutured, and the mice were allowed to recover under observation.

### Injection of Tumor Cells via the Portal Vein

To mimic metastatic spread, selected PDAC cell lines were injected into the portal vein following a refined surgical protocol established in our laboratories.^15^ This procedure was meticulously performed, ensuring minimal stress and discomfort to the animals.

After surgery, the mice received postoperative care, including analgesia to manage pain. A detailed video of the surgical procedure can be viewed through the provided link: https://drive.google.com/file/d/1LfMdqzatZYnvMqGPSf3_01m381J634bp/view.

After a minimum 48-h acclimatization period, the recipient mice were sedated with an intraperitoneal injection of 100 mg/kg ketamine/xylazine in preparation for the surgical procedure. An incision approximately 2 cm long was made just below the xyphoid process. The intestines were gently moved to the left side of the peritoneal cavity and placed on moist gauze to expose the portal vein. The cells, suspended in 100 μl of saline within a 1.5-ml plastic tube, then were loaded into a 1-ml plastic syringe equipped with a 29-gauge 12.5-mm needle. This needle was carefully inserted into the visible portal vein to administer the cell suspension.

After injection and needle withdrawal, direct pressure was applied to halt any bleeding. A bleeding score was established as follows: 0 (no bleeding), 0.5 (minor bleeding: ~50 μL, half a cotton swab), 1 (moderate bleeding: ~100 μL, one full cotton swab), 1.5 (steady bleeding: ~150 μL, 1½ cotton swabs), and 2 (severe bleeding: ~ 200 μL, two full cotton swabs). If the bleeding score reached 2 or more (>25 % of the mouse’s total blood volume), sterile water for injections was used to wash the intraperitoneal space to prevent peritoneal dissemination.

Subsequently, the internal organs were carefully returned to their original positions, and the peritoneal cavity and abdominal incision were securely closed. The mice then were gradually brought out of anesthesia. To manage postoperative pain, an analgesic (carprofen 5 mg/kg) was administered subcutaneously for 2 days after surgery.

### Derivation of the DT6606 lm Line

A metastatic tumor was harvested from the liver of a C57BL/6 mouse that had been injected previously with DT6606 cells into the portal vein, leading to the development of liver metastases. The excised tumor tissue was initially rinsed with ×1 phosphate-buffered saline (PBS; Sigma-Aldrich, St. Louis, MO, USA) on a petri dish before being sectioned into smaller fragments using a sterile surgical blade. These fragments then were transferred to a 50-ml falcon tube filled with an enzymatic digestion solution (comprising 255 U/mg collagenase and 25 μl DNAse, Sigma-Aldrich, St Louis, MO) and incubated at 37 °C for 2 h with intermittent gentle agitation.

After incubation, the digestion was halted by adding 5 ml of RPMI medium containing 10 % FBS (Thermo-fisher, Waltham, MA), followed by centrifugation at 1200 rpm for 8 min. The supernatant was removed, and the resultant pellet was resuspended in 2 ml of RPMI medium supplemented with 2 % FBS. The suspension then was passed through a 40-μm cell strainer (Sigma-Aldrich, St Louis, MO) and cultured in a six-well plate. The culture medium was refreshed daily for a week to eliminate any remaining cell debris.

### Preclinical Mouse Imaging: Seven-Tesla MR

For the acquisition of MRI, the mice were subjected to inhalational anesthesia and positioned on a specialized apparatus designed for temperature regulation to avert hypothermia, with their breathing rate and body temperature continuously monitored (SA Instruments, Stony Brook, New York, NY, USA). A liver-specific contrast medium, Gd-EOB-DTPA (also known as gadoxetic acid, 0.05 lmol/g of body weight; Bayer Schering Pharma, Berlin, Germany), was administered to enhance visualization of liver lesions.

For imaging, the animals were positioned prone on the scanner bed. Imaging was performed with a seven-Tesla horizontal MRI scanner (Bruker, BioSpec 70/30 USR, Paravision 5.1, Germany), which is equipped with a powerful gradient system (amplitude of 450/675 mT/m, slew rate of 3400/4500 T/m/s, and a rise time of 140 ms) and a mouse-specific volumetric body coil. Next, T2-weighted axial images with fat saturation (TurboRARE-T2: TR, 3700 ms; TE, 30 ms; spatial resolution, 0.133 × 0.08 mm/pixel; slice thickness, 0.6 mm; no gap; averages, 6) were captured, covering the entire abdominal area. Analysis of the MRI data was performed using MIPAV software (NIH, Bethesda, MD, USA) by radiologists highly experienced in both clinical and preclinical imaging evaluations.

### Histology and Immunohistochemistry

For histologic and immunohistochemical analysis, liver samples were harvested, preserved in zinc-formalin solution (Sigma-Aldrich, St. Louis, MO, USA), and subsequently embedded in paraffin. Thin sections (5 μm) were prepared and subjected to staining processes, including hematoxylin and eosin (H&E), anti-mouse cytokeratin 19 (CK19) rabbit monoclonal antibody (mAb) (Abcam, Cambridge, UK), anti-mouse E-cadherin rabbit mAb (Cell Signaling Technology, Danvers, MA, USA), anti-mouse Trp53 rabbit polyclonal antibody (Ab) (Novocastra, Newcastle upon Tyne, UK), Sirius Red (Direct Red 80; Sigma-Aldrich, St. Louis, MO, USA), and Alcian Blue (8GX; Sigma-Aldrich, St. Louis, MO, USA). Immunohistochemical staining was performed using the EXPOSE Rabbit-Specific HRP/DAB (horseradish peroxidase/3,3'-diaminobenzidine) detection kit following the manufacturer's instructions (Abcam, Cambridge, UK). These slides then were reviewed by a skilled pathologist. Image capture was facilitated by the Aperio AT2 digital pathology scanner (Leica Biosystems, Milano, IT).

### Statistical Analysis

Data are presented as mean ± standard error (SE). Survival rates were analyzed with Kaplan–Meier curves, and differences were assessed using the log-rank test. A *p* value of 0.05 or lower was deemed to indicate statistical significance. All statistical evaluations were performed using SPSS version 13.0 (SPSS Inc., Chicago, IL, USA). Graphic representations were created with GraphPad Prism version 9 (GraphPad Software, La Jolla, California, USA).

## Results

### Assessing Metastasis and Survival in an Orthotopic PDAC Model

Initially, we developed an orthotopic model of PDAC using 8-week-old male C57BL/6 mice by directly injecting PDAC cells into the pancreas. The chosen DT6606 cell line, derived from LSL-KrasG12D/+; Pdx-1-Cre (KC) mice of a C57BL/6 background, was used for these experiments. This study evaluated the survival of 20 mice, which were administered varying doses of DT6606 cells ranging from 5 × 10^4^ to 2 ×10^6^ cells per mouse, with each dosage group comprising four mice. The results uniformly showed that all mice succumbed to the tumor within an 11-week period, resulting in an average survival time of 78.2 ± 4.45 days (Table 1). This reflected the rapid and aggressive nature of the tumor growth prompted by orthotopic implantation during a short span.

**Table 1:** Estimated means and medians of survival time (days) for mice receiving DT6606 cells

VAR00001	Mean				Median			
	Estimate	Std. error	95% CI		Estimate	Std. error	95% CI	
			Lower bound	Upper bound			Lower bound	Upper bound
2,000,000 cell/mouse	68,500	9700	49,489	87,511	54,000	15,500	23,620	84,380
1,000,000 cell/mouse	63,000	5802	51,627	74,373	60,000	11,000	38,440	81,560
500,000 cell/mouse	76,500	7665	61,477	91,523	67,000	14,000	39,560	94,440
100,000 cell/mouse	90,750	5935	79,117	102,383	81,000	10,000	61,400	100,600
50,000 cell/mouse	92,250	13,369	66,047	118,453	84,000	17,500	49,700	118,300
Overall	78,200	4454	69,471	86,929	80,000	5217	69,774	90,226

*Estimation is limited to the largest survival time if it is censored

**Fig. 1:**
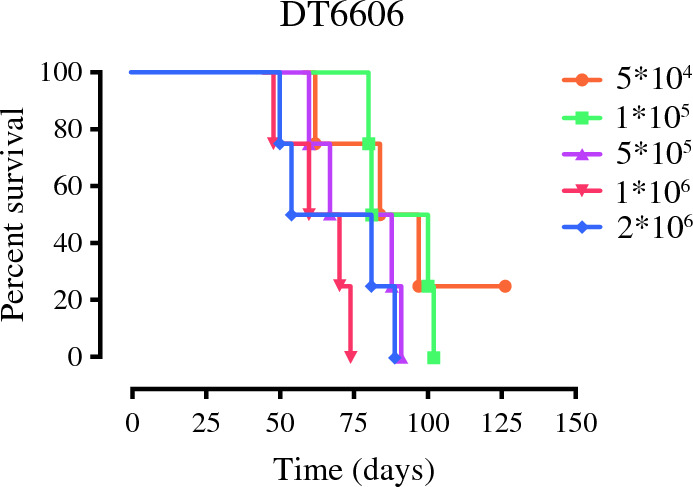
Overall survival of mice with orthotopically induced pancreatic ductal adenocarcinoma (PDAC). The graph represents the Kaplan-Meier plot of overall survival for the mice that received five different doses of DT6606 cell line. The *p* value was estimated by the log-rank test

Post-mortem examinations failed to identify any instances of liver metastasis. A notable aspect was the evident dose-dependent correlation between the cell quantity used for initiating tumor growth and the survival rates observed in the mice, as illustrated in Fig. 1. Moreover, it is important to note that although this model facilitated the synchronous onset and progression of the primary tumor, the rapid expansion of the primary tumor and the resultant mortality, primarily due to peritoneal dissemination, did not allow adequate time for the development of metastases, highlighting a limitation in the model's capacity to mimic the metastatic stage of PDAC. Similar results in a small setting of mice were obtained also with the K8484 cell line (data not shown).

### Induction of Liver Metastases Through Portal-Vein Injection: Dose-Response Relationship in Metastatic Penetrance

To develop a robust and clinically relevant PDAC metastasis model, we used portal-vein injection in 8-week-old male C57BL/6 mice to directly induce liver metastases. This approach is crucial for the investigation of PDAC’s metastatic journey to the liver, a frequent target for disease progression in humans.

For this experiment, we used both the DT6606 and K8484 cell lines. It is worth noting that the DT6606 cell line is derived from LSL-KrasG12D/+; Pdx-1-Cre (KC) mice, and that the K8484 line comes from LSL-KrasG12D/+; LSL-Trp53R172H/+; Pdx-1-Cre (KPC) mice. These cell lines showcase different genetic profiles that reflect the diverse levels of aggressiveness and potential for metastasis in PDAC, thus offering a detailed perspective on tumor dynamics and spread in a living system.

**Fig. 2:**
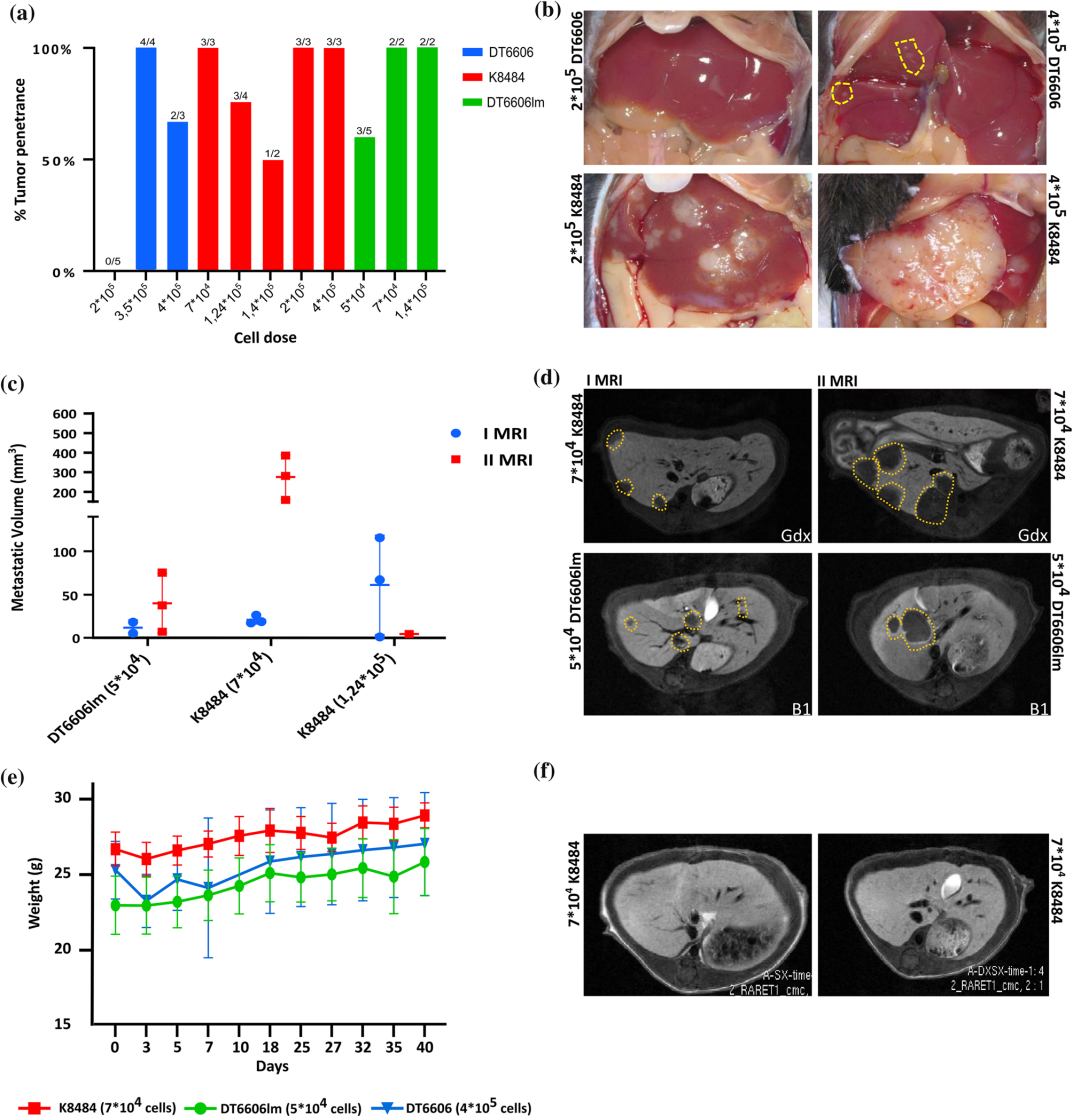
Evaluation of metastatic development in the liver of mice receiving K8484, DT6606, and DT6606lm cell lines via intraportal injection. **A** The histograms show the tumor penetrance (%) achieved by injecting different doses of the three cell lines. The number of mice with tumors among the total infused mice is represented. **B** Representative images showing the explanted livers of two mice in which metastatic lesions developed after injection with DT6606 and K8484 cells. Livers were collected after the mice were killed on day 21 after intraportal injection. **C** The graph shows the volume (mm^3^) of liver metastases that developed in mice infused with DT6606lm (dose 5 × 10^4^) and K8484 (doses 7 × 10^4^ and 1.24 × 10^5^). The metastatic lesions were detected and measured on two MRIs on days 21 and 36 after intraportal injection. **D** Representative images of the T1 liver sections captured on the MRIs showing liver metastases in two mice infused with DT6606lm (dose 5 × 10^4^) and K8484 (dose 7 × 10^4^). **E** The graph illustrates the weights trend in mice previously infused with the three cell lines. **F** Representative images of the T1 liver sections captured on MRI showing the livers of two female mice previously infused with K8484 cells

In the investigation of dose-dependent effects on metastatic development, cell injections were administered across a spectrum from 7 × 10^4^ to 4 × 10^5^ cells per mouse, to evaluate the correlation between cell dose and metastatic incidence. Mice inoculated with K8484 cells achieved a consistent 100 % rate of tumor take at cell doses of 7 × 10^4^, 2 × 10^5^, and 4 × 10^5^ (Fig. 2A). Examination of the livers 21 days after injection showed extensive metastatic involvement in mice treated with 2 × 10^5^ and 4 × 10^5^ K8484 cells, highlighting a significant metastatic burden at these higher doses (Fig. 2B).

In contrast, the administration of DT6606 cells resulted in an absence of liver metastases at the 200,000-cell dose, with only a sparse metastatic presence observed at doses of 3.5 × 10^5^ and 4 × 10^5^ per mouse (Fig. 2A, B). These findings illustrate a clear dose-response relationship, particularly pronounced in the K8484 cell line, underscoring its higher metastatic potential compared with DT6606 cells.

To improve the metastatic efficiency of the DT6606 cell line, we derived a new variant, named DT6606lm, from a metastatic liver lesion in a mouse previously injected via the portal vein with the original DT6606 cells (refer to the Materials and Methods section). This modification was intended to increase the line's proficiency in generating liver metastases. In a considerable number of mice injected with DT6606lm, tumors developed, with a 100 % incidence rate observed at cell doses of 7 × 10^4^ and 1.4 × 10^5^, and a 60 % incidence rate observed at a dose of 5 × 10^4^ (Fig. 2A). However, mice that received 7 × 10^4^ and 1.4 × 10^5^ DT6606lm cells died by day 21 after injection. Post-mortem examinations of these mice showed significant metastatic liver involvement, characterized by jaundice, discoloration of the gallbladder, and obstruction of the bile duct (data not shown).

### Establishing Conditions for In Vivo MRI Monitoring of Liver Metastases via Portal-Vein Injection

Establishing a model for monitoring metastatic progression is pivotal for assessing the efficacy of treatments. This model needs to provide a sufficient window for administering therapeutic interventions and the capability to evaluate their effects using imaging techniques. To achieve this objective, increasing doses of DT6606lm or K8484 cells were administered to mice through the portal vein, enabling the assessment of liver metastasis via MRI at two critical junctures: 21 and 36 days after cell injection. Sequential MRI scans demonstrated that doses of 5 × 10^4^ DT6606lm cells and 7 × 10^4^ K8484 cells resulted in steady metastatic growth by day 21, which then escalated by day 36, as depicted in Fig. 2C, D. However, higher doses proved detrimental for the model's objectives. Specifically, mice injected with 7 × 10^4^ and 1.4 × 10^5^ DT6606lm cells succumbed by day 21 after injection. Autopsies of these mice showed extensive metastatic liver damage, evidenced by jaundice, a darkened gallbladder, and bile duct obstruction (details not shown). Similarly, mice receiving 1.4 × 10^5^ K8484 cells displayed significant metastatic lesions on the initial MRI, with two thirds dying before the second MRI could be performed. Considering the established doses, the tolerability of the procedure was assessed through continuous weight-monitoring.

After tumor induction, the weight of the animals was regularly tracked, showing a rapid recovery after an initial brief period of discomfort. This quick rebound after surgery indicated the procedure's overall tolerability (Fig. 2E).

Additionally, the influence of sex on the model's effectiveness was examined. Specifically, to determine whether sex has an impact on tumor development, twelve 8-week-old female C57BL/6 mice were injected with the K8484 cell line at a dosage of 7 × 10^4^ cells per mouse, mirroring the procedure used with male mice. Interestingly, none of these female mice exhibited metastatic liver lesions when evaluated by MRI on day 21 after injection (Fig. 2F).

### Histologic Evaluation of Liver Metastases After Tumor Induction

**Fig. 3:**
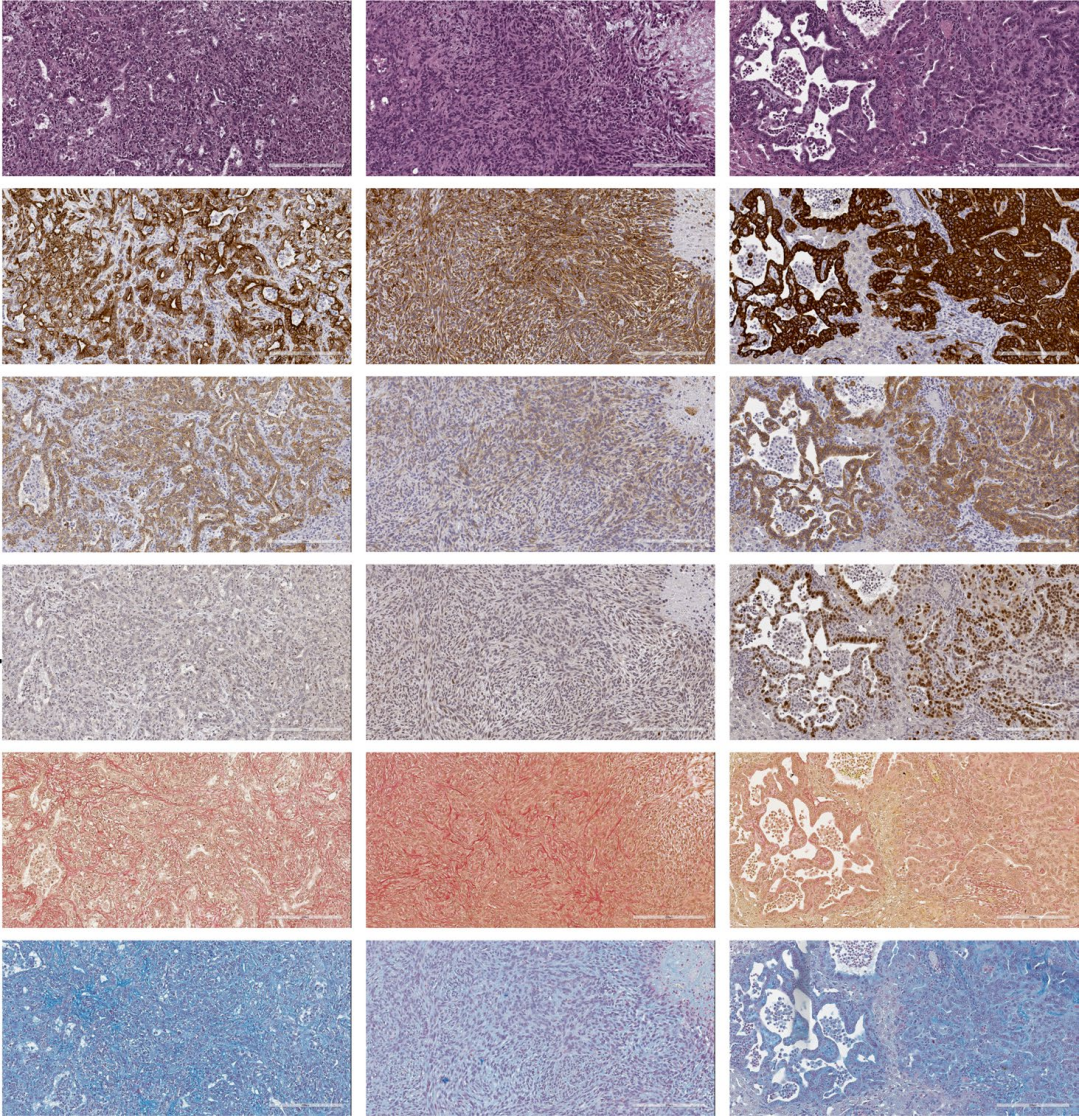
Histologic and immunohistochemical characterization of liver metastases in mice with surgically induced liver metastases. The panels display representative histologic images of the liver serial sections in mice. White bars: 200 μm. For **A** DT6606-derived liver metastases, **B** DT6606lm-derived liver metastases, and **C** K8484-derived liver metastases are shown: the hematoxylin and eosin (H&E) staining, the staining for CK19, the E-cadherin membranous staining, the Trp53 nuclear expression, the Sirius Red staining of the collagen deposition, and the Alcian Blue staining of stroma indicate hyaluronan deposition

The mice were killed 22 days after tumor induction, and their livers were extracted for subsequent analysis. The metastatic lesions in the livers, induced by the DT6606, DT6606lm, and K8484 cell lines, were subjected to immunohistochemical analysis to delineate their characteristics. Lesions originating from DT6606 displayed widespread and strong CK19 expression, with E-cadherin localized to the membrane within the glandular structures. There was an absence of nuclear TP53 expression and minimal extracellular matrix presence. Overall, metastases from DT6606 were characterized as poorly differentiated, with about half exhibiting glandular/cribriform adenocarcinoma features (Fig. 3A). Lesions from DT6606lm also exhibited widespread CK19 expression, with E-cadherin present on the membrane of the glandular but not the sarcomatoid components. Minimal TP53 staining was observed, and a slight fibrotic stroma within the tumor was noted. Consequently, DT6606lm metastases were identified as poorly differentiated, with a small portion of glandular structures and distinct sarcomatoid regions (Fig. 3B). Finally, metastases originating from K8484 showed strong and widespread CK19 and TP53 expression, with uniform E-cadherin membrane localization and scant fibrotic stroma. These metastases were moderately differentiated, closely resembling human metastases in their predominantly glandular structure (Fig. 3C).

## Discussion

Considering the inefficacy of conventional therapies in addressing metastatic disease, treatments to treat PDAC-derived metastasis successfully constitute an unmet clinical need.^16^ The development of a preclinical model enabling the treatment of metastases would be extremely valuable in this context.

The advancement of preclinical models has significantly contributed to our understanding of PDAC, particularly in terms of tumor development, the tumor–microenvironment interaction, and the dynamics of tumor-infiltrating immune cells.^17^ These models serve as invaluable tools for exploring novel therapeutic strategies and drug-testing. In this domain, GEMMs of pancreatic cancer, which incorporate human PDAC-specific genetic mutations into the mouse genome, represent a step forward.^18,19^ However, certain limitations are clear, such as the inconsistent tumor growth rates and extended latency periods frequently observed in GEMMs.^20^ These issues are particularly problematic for time-sensitive research and the assessment of therapeutic strategies. The development and progression of primary tumors in GEMMs are not strictly age-dependent, leading to a lack of consistent timing in PDAC development. This inconsistency significantly restricts the usefulness of GEMMs for experimental research, notably affecting the ability to establish a specific time frame for initiating and evaluating treatment interventions.

The inherent limitations encountered in the development of primary tumors within GEMMs might be partially alleviated through the introduction of an age-independent tumor-staging system. As delineated in our prior work,^10^ this innovative approach would harness the capabilities of advanced preclinical imaging technologies, such as seven-Tesla magnetic resonance (MR) and ultrasound (US). These methods promise a uniform framework for assessing tumor progression, decoupled from the chronological age of the subject mice. However, the effort to standardize a liver metastasis model presents a more formidable challenge. Furthermore, the erratic metastatic evolution observed in the KPC and KC models significantly undermines their efficacy for preclinical testing, posing substantial obstacles to their application in the advancement of translational research efforts.^4^

In this study, we established an effective transplantation model for PDAC to induce metastatic dissemination in the liver. The presented model offers several advantages in addressing the limitations and gaps associated with GEMMs for PDAC, particularly those related to the sporadic progression to metastasis observed in the KPC and KC models. First, unlike the sporadic and unpredictable metastasis in traditional GEMMs, this model uses a heterotopic transplantation method, with direct injection of tumor cells into the circulation. This approach more reliably simulates the steps of cancer dissemination, extravasation, and colonization, specifically targeting the liver to induce metastatic growth.^11^ This systematic approach provides a controlled environment for studying the metastatic process in detail.

Second, through dose-response analysis and MRI characterization, the model enables precise control over the number and size of metastatic lesions in the liver. This allows for a uniform distribution of metastatic lesions to be generated across experiments, facilitating consistent and reproducible results that are difficult to achieve with GEMMs due to their inherent variability in tumor growth and metastasis.

Third, the model's capability to induce limited and homogeneous liver metastases within a defined time frame enables the effective scheduling of therapeutic interventions. This aspect is particularly beneficial for preclinical trials, in which timing and response to treatment are critical parameters.

Fourth, even if at a different grade, it is possible to mimic human PDAC lesions.^21,22^ For example, the use of K8484 cells ensures that the induced tumors in mice closely resemble the histopathologic characteristics of human PDAC, including Trp53+ tumor cell density and glandular architecture. This enhances the translational relevance of the model, making it more applicable for the study of human PDAC and the testing of human-specific therapies.

Fifth, despite the invasive nature of the intraportal injection technique, the model is shown to be feasible and does not adversely affect the overall health status of the animals. This ensures that the model can be used without causing undue harm or distress, adhering to ethical considerations in animal research.

Sixth, no peritoneal metastases were observed in the portal-vein-injection group, which can be attributed to precise surgical execution minimizing significant bleeding. This outcome is beneficial because it allows for the assessment of survival based strictly on liver disease progression, without interference from other disease burdens. In contrast, the orthotopic model showed numerous peritoneal metastases, likely linked to the intraperitoneal positioning of the pancreas in mice and possible leakage of cells during injection.

The model presented in this study, although effective for studying certain aspects of metastatic development, does come with limitations that warrant consideration. First, the successful implementation of the portal-vein injection technique requires significant microsurgical expertise, restricting its use to facilities with such capabilities. An alternative could be the ultrasound-guided portal-vein injection method. Although not used in our study, this technique might reduce the need for surgical interventions. However, it presents several challenges, as outlined in previous studies, including difficulties maintaining needle alignment, potential mis-injection, and difficulties identifying the portal vein in fatty environments.^23^ In contrast, our surgical method allows for direct visualization and clamping of the portal vein, ensuring accurate needle placement and reducing hemorrhage risks. Although more invasive, our approach is well-tolerated by mice, with effective pain management and rapid recovery after operation.

Second, this model does not incorporate a preconditioning phase for the primary tumor, which can be crucial for understanding the influence of tumor environment on metastasis. Most notably, the model bypasses the initial phases of the metastatic process, including tumor cell detachment, invasion, and migration through the bloodstream. Consequently, although this model is well-suited for examining tumor cell engraftment and the subsequent development of metastases, it does not provide insights into the early steps of the metastatic cascade. This specificity makes it an excellent tool for focused studies but less applicable for investigations of the complete spectrum of metastatic progression.

Finally, another limitation of our study was the necessity of using male recipients. This requirement may restrict the generalizability of our findings across different biologic contexts and could potentially influence the interpretation of the tumor behavior in broader clinical scenarios. The variation in liver metastasis between male and female mice could be explained by several intrinsic and extrinsic factors, but not by minor antigen mismatches related to the Y chromosome, considering the female derivation of the K8484 cell line. The differences in tumor behavior between the sexes are likely due to hormonal differences (e.g., levels of estrogen and testosterone) and variations in immune responses. Furthermore, as documented in our previous publications,^24^ the ability of the K8484 line to engraft in non-liver sites in both male and female mice highlights the liver's distinct sensitivity to these sex-related differences. This hypothesis is supported by recent research indicating significant gender disparities in cancer, notably in the KPC pancreatic cancer model.^25^ Studies specifically point to the tissue-inhibitor role of metalloproteinases 1 (TIMP1), which is up-regulated in males and linked to reduced survival and increased liver metastasis, suggesting a complex, sex-specific biologic mechanism that could affect metastatic patterns and outcomes.

In summary, the model proposed in this study addresses the critical gap in PDAC research by providing a reliable, controllable, and ethically sound platform for the study of liver metastasis. It overcomes the limitations of traditional GEMMs, offering a valuable tool for the advancement of therapeutic strategies against PDAC-derived metastasis.

### Supplementary Information

Below is the link to the electronic supplementary material.

Supplementary file1 (MP4 14045 kb)
